# Copper as a target for prostate cancer therapeutics: copper-ionophore pharmacology and altering systemic copper distribution

**DOI:** 10.18632/oncotarget.9245

**Published:** 2016-05-09

**Authors:** Delphine Denoyer, Helen B. Pearson, Sharnel A.S. Clatworthy, Zoe M. Smith, Paul S. Francis, Roxana M. Llanos, Irene Volitakis, Wayne A. Phillips, Peter M. Meggyesy, Shashank Masaldan, Michael A. Cater

**Affiliations:** ^1^ Centre for Cellular and Molecular Biology, School of Life and Environmental Sciences, Deakin University, Burwood, Victoria, Australia; ^2^ Research Division, Peter MacCallum Cancer Centre, East Melbourne, Victoria, Australia; ^3^ Sir Peter MacCallum Department of Oncology, the University of Melbourne, Parkville, Victoria, Australia; ^4^ Centre for Chemistry and Biotechnology, School of Life and Environmental Sciences, Deakin University, Waurn Ponds, Victoria, Australia; ^5^ The Florey Institute of Neuroscience and Mental Health, the University of Melbourne, Parkville, Victoria, Australia; ^6^ Department of Pathology, the University of Melbourne, Parkville, Victoria, Australia

**Keywords:** copper, ionophore, prostate cancer, Atp7b, TRAMP

## Abstract

Copper-ionophores that elevate intracellular bioavailable copper display significant therapeutic utility against prostate cancer cells *in vitro* and in TRAMP (Transgenic Adenocarcinoma of Mouse Prostate) mice. However, the pharmacological basis for their anticancer activity remains unclear, despite impending clinical trails. Herein we show that intracellular copper levels in prostate cancer, evaluated *in vitro* and across disease progression in TRAMP mice, were not correlative with copper-ionophore activity and mirrored the normal levels observed in patient prostatectomy tissues (Gleason Score 7 & 9). TRAMP adenocarcinoma cells harbored markedly elevated oxidative stress and diminished glutathione (GSH)-mediated antioxidant capacity, which together conferred selective sensitivity to prooxidant ionophoric copper. Copper-ionophore treatments [Cu^II^(gtsm), disulfiram & clioquinol] generated toxic levels of reactive oxygen species (ROS) in TRAMP adenocarcinoma cells, but not in normal mouse prostate epithelial cells (PrECs). Our results provide a basis for the pharmacological activity of copper-ionophores and suggest they are amendable for treatment of patients with prostate cancer. Additionally, recent *in vitro* and mouse xenograft studies have suggested an increased copper requirement by prostate cancer cells. We demonstrated that prostate adenocarcinoma development in TRAMP mice requires a functional supply of copper and is significantly impeded by altered systemic copper distribution. The presence of a mutant copper-transporting Atp7b protein (tx mutation: A4066G/Met1356Val) in TRAMP mice changed copper-integration into serum and caused a remarkable reduction in prostate cancer burden (64% reduction) and disease severity (grade), abrogating adenocarcinoma development. Implications for current clinical trials are discussed.

## INTRODUCTION

Prostate cancer is a major cause of morbidity and mortality among elderly men worldwide and is rapidly becoming more prevalent as life expectancy increases [1, 2]. Early detection is paramount, and treatment regimes are disease stage-dependent and can include combinations of radical prostatectomy, brachytherapy (implanted radioactive seeds) and androgen deprivation (hormone therapy). Unfortunately, these therapies are often not curative and the majority of patients relapse into castration-resistant (hormone-refractory) disease. While attention is still focused primarily on developing androgen deprivation, a number of additional pharmacological targets have emerged (e.g. protein tyrosine kinases [3], mitochondrial metabolism [4] or prostate-specific membrane antigen [5]), highlighting the growing consensus that alternative therapies for prostate cancer are needed [6]. We recently established that prostate cancer cells, both *in vitro* and in the orthotopic TRAMP mouse model, are remarkably sensitive to a family of lipophilic compounds categorized as copper-ionophores [7–9]. Ionophores bind and transport specific metal(s) indiscriminately into cells, often allowing the ions to become bioavailable (exchangeable) [8, 10, 11]. Importantly, copper is a potent prooxidant and excess causes the generation of cytotoxic reactive oxygen species (ROS) in cells [9, 12]. The differential response between normal (healthy) and cancerous cells to select copper-ionophores is the basis for their development against a variety of cancer types, including melanoma and breast cancer [13–15]. Nevertheless, the pharmacological mechanism responsible for their selective toxicity against cancerous cells remains unclear.

Physiological copper interchanges between oxidized cupric (Cu^II^) and reduced cuprous (Cu^I^) states, enabling it to serve as a rate-limiting cofactor for enzymes fundamental for cellular growth and development (reviewed in [9]). Cellular acquisition and utilization of prooxidant copper is tightly regulated by molecular transporters and chaperones to prevent deleterious ROS production while satiating cuproenzyme metallation [9]. Nevertheless, several reports describe copper levels being characteristically elevated (2-6 fold) in prostate cancer patient cell lines *in vitro* [7, 16, 17] and in xenograft mouse models [17, 18], suggesting that patients might harbour elevated intratumoral copper. Raised intracellular ROS is a well-defined feature of human prostate cancer and clinical studies have unequivocally confirmed a role for oxidative stress in the development and progression of this disease [19–22]. Therefore, it has been postulated that elevated endogenous copper may predispose prostate cancer cells to copper-ionophore sensitivity, by possibly underpinning a heightened state of oxidative stress [8, 14, 17, 23]. However, copper ions can exert toxicity through a myriad of mechanisms, including protein iron-sulfur cluster interference, proteasome inhibition and by displacing functional metals (e.g. zinc and iron) from metalloproteins [9, 24, 25]. Furthermore, we recently established that only a small subset of prostate cancer patients actually harbour elevated intratumoral copper levels, irrespective of their disease stage (Gleason Score 7 or 9) [26]. Therefore, clarification on the importance of intratumoral copper for the pharmacological activity of copper-ionophores is required.

The anticancer activity of bis(thiosemicarbazone) copper ligands was established in numerous *in vitro* and *in vivo* studies mid last century [27–29], originating with the demonstration that H_2_gts [glyoxalbis(thiosemicarbazone)] inhibited sarcoma growth in Swiss brown mice [29]. We recently established that Cu^II^(gtsm) [glyoxalbis(*N*^4^-methylthiosemicarbazonato)Cu^II^] significantly reduced prostate cancer burden (~70%) and severity (lesion grade) in the orthotopic TRAMP mouse model [8]. Pharmacokinetic analyses in mice confirmed that Cu^II^(gtsm) did not exchange coordinated copper with other divalent metals *in vivo* [8]. Mechanistically, Cu^II^(gtsm) undergoes intracellular reduction causing copper (Cu^I^) to dissociate into a bioavailable (prooxidant) pool. Remarkably, the resultant ligand (H_2_gtsm) continues to re-coordinate and redistribute accessible copper [8]. This property renders Cu^II^(gtsm) highly toxic toward human prostate cancer cell lines (e.g. PC3, DU145, LNCap), while normal cells (e.g. human primary prostate epithelial cells) remained refractory [8]. The anticancer activity of Cu^II^(gtsm) was copper-dependent, positively correlated with milieu copper level and could be abrogated with copper chelation [tetrathiomolybdate (TTM)] [8]. Stefani and colleagues (2015) more recently demonstrated that Cu^II^(gtsm) generates intracellular ROS and again validated the requirement for copper for its anticancer activity [23]. Two structurally unrelated copper-ionophores, disulfiram [1-(diethylthiocarbamoyldisulfanyl)-*N*,*N*-diethyl-methanethioamide)] and clioquinol (5-chloro-7-iodo-8-quinolinol), likewise display selective pharmacological activity against prostate cancer *in vitro* and in mouse models [8, 16, 17]. Analogous to Cu^II^(gtsm), their anticancer potency correlated with an intrinsic ability to release coordinated copper under the reductive intracellular environment [8]. Disulfiram was identified in a screen for prostate cancer therapeutics and was subsequently evaluated in clinical trials on patients with non-metastatic recurrent prostate cancer (500 mg/day) [30]. Disulfiram failed to demonstrate clinical activity in these patients, but its pharmacokinetics in relation to copper was not evaluated and its apo (copper-free) structure was administered. Disulfiram, and clioquinol, absolutely require coordinated copper for anticancer activity [7, 8]. Another clinical trial evaluating disulfiram in the setting of parenteral copper supplementation is in the pipeline [17].

Clinical trials of copper-ionophores for prostate cancer treatment are clearly outpacing our understanding of their mechanism of action. In this study, we aimed to delineate the role of elevated intratumoral copper and ROS production and establish how copper-ionophores are selectively toxic toward prostate cancer cells. Additionally, recent *in vitro* and mouse xenograft studies have suggested an increased copper requirement by prostate cancer cells [7, 8, 16–18, 31]. Therefore, we sought to determine the importance of copper in prostate cancer development and progression in the orthotopic TRAMP mouse model.

## RESULTS

### Copper homeostasis is maintained during prostate cancer progression in TRAMP mice

**Figure 1:**
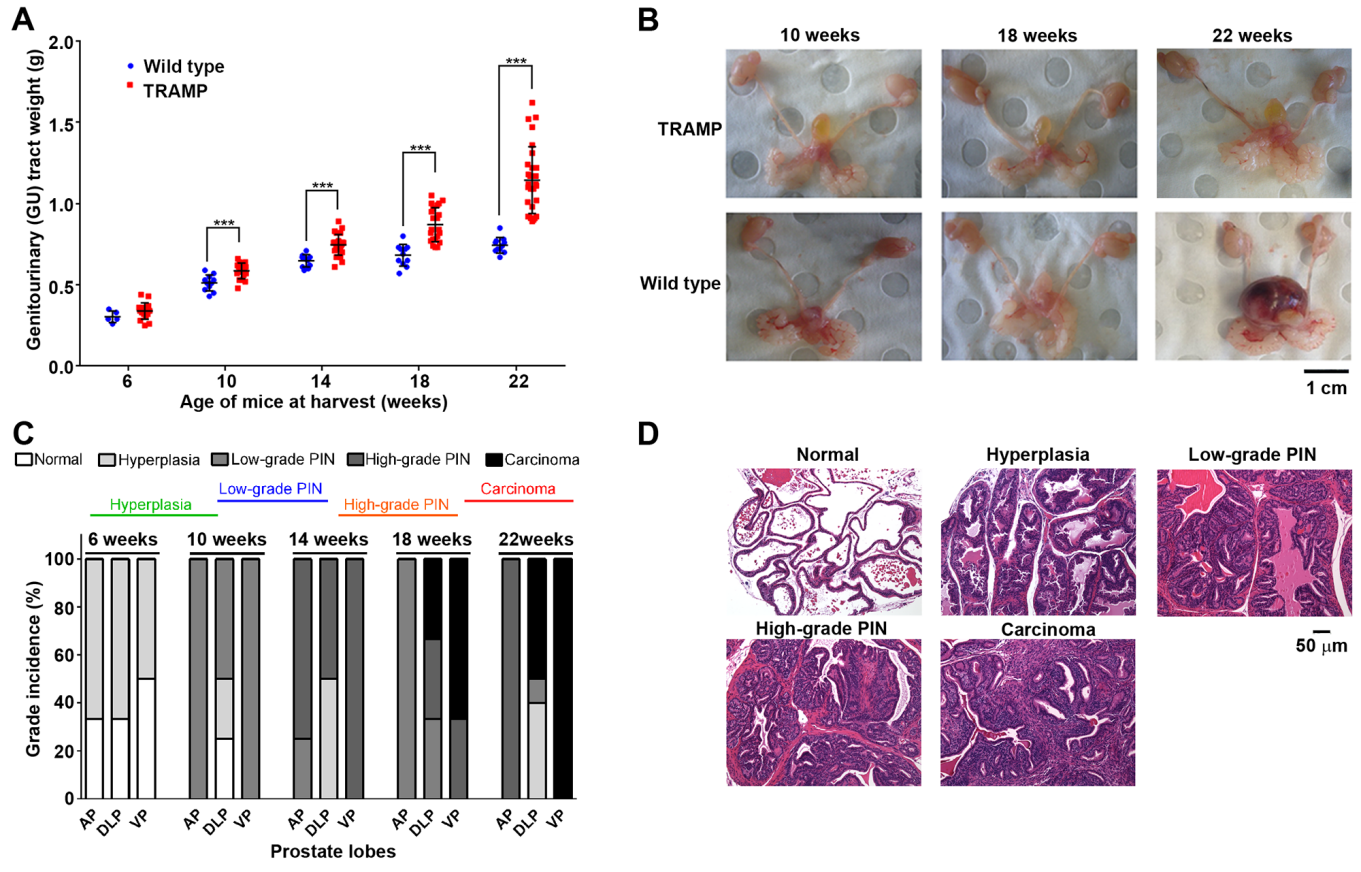
Prostate cancer develops uniformly in the TRAMP mouse model **A.** Progressive cancer burden in TRAMP mice monitored by the cumulative weight of the genitourinary (GU) tract (includes prostate, seminal vesicles, testicles and empty urinary bladder). GU tracts were weighed from both wild type and TRAMP mice at the indicated ages (6-22 weeks) and normalised to respective mouse body weights (n=5-15 at each age). Horizontal black lines represent the mean GU tract weight at the indicated age. **B.** Representative photographs of harvested GU tracts from wild type and TRAMP mice at 10, 18 and 22 weeks of age. **C.** Histological examination of prostate lobes [anterior prostate (AP), dorsolateral prostate (DLP) and ventral prostate (VP)] with hematoxylin and eosin (H&E) staining, verifying disease grade in TRAMP mice at the indicated ages (6-22 weeks) (n=5 at each age). The most advanced proliferative lesion in each lobe signified the grade of disease. **D.** Representative H&E-stained sections displaying grades of prostate disease in TRAMP mice from hyperplasia, low-grade PIN, high-grade PIN, adenocarcinoma and invasive adenocarcinoma. H&E-stained normal prostate was obtained from wild type mice. (***p < 0.001).

**Figure 2:**
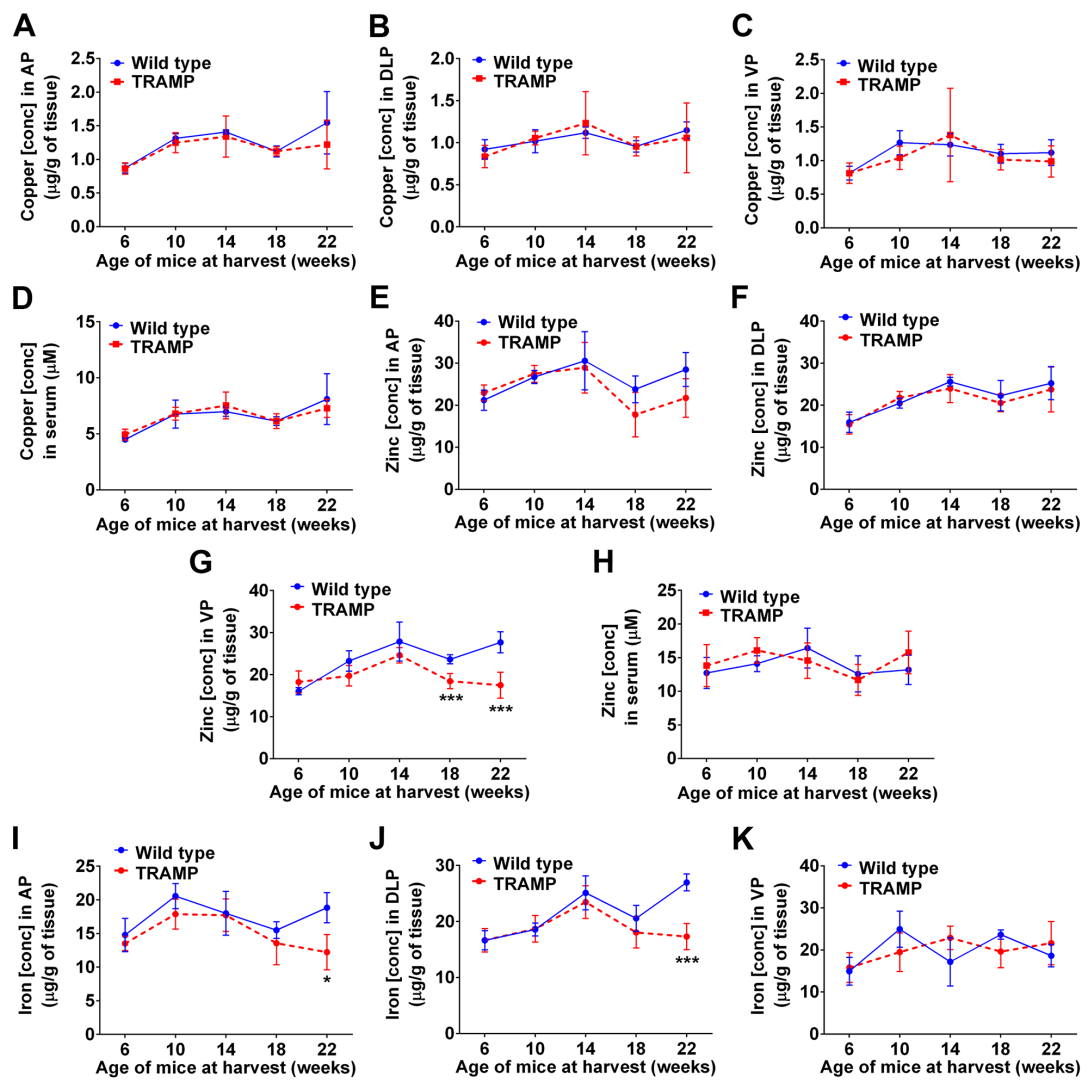
Metal levels in prostate tissue and serum during cancer development in TRAMP mice Inductively coupled plasma mass spectrometry (ICP-MS) was used to determine whether copper, zinc or iron concentrations change in prostate lobes and sera of TRAMP mice throughout disease progression. **A-D.** Copper concentrations in anterior prostate (AP), dorso-lateral prostate (DLP), ventral prostate (VP) and serum from both wild type and TRAMP mice at the indicated ages (6-22 weeks). Zinc **E-H.** and iron **I-K.** concentrations are also shown. Results represent mean ± STDEV (bar) and are shown as either μg/g wet weight for tissues (n=5-10 at each age) or μM for serum (n=10-17 at each age). (*p < 0.05; ***p < 0.001).

We previously demonstrated that prostate cancer cells *in vitro* and in the orthotopic TRAMP mouse model are highly sensitive to copper-ionophores that increase intracellular bioavailable copper [7, 8]. We, and others, postulated that endogenous elevated intracellular copper predisposed the cancer cells to sensitivity, by possibly underpinning a heightened state of oxidative stress [8, 14, 17, 23]. However, we recently established that only a small subset of prostate cancer patients' harbour elevated intratumoral copper [26] and thus sought to clarify whether copper is indeed the critical, and targetable, factor. To address this question, we surveyed copper levels during prostate cancer development in TRAMP mice (Figure 1 & 2), having previously obtained significant copper-ionophore [Cu^II^(gtsm)] utility in this model [8]. Heterozygous male TRAMP mice develop prostate adenocarcinoma through the expression of the SV40 large T antigen (Tag), which is driven by the prostate epithelial specific promoter, probasin. Probasin is androgen-driven and therefore both Tag expression and cancer initiation are developmentally regulated in the mice, beginning at 6-weeks of age [32]. Disease development in TRAMP mice is well-characterized [8, 32–35], with progressive cancer burden monitored by the cumulative weight of the genitourinary (GU) tract (includes prostate, seminal vesicles, testicles and empty urinary bladder) (Figure 1A & 1B). Histological examination of prostate lobes [anterior prostate (AP), dorsolateral prostate (DLP) and ventral prostate (VP)] with hematoxylin and eosin (H&E) staining, verified that prostate cancer develops uniformly in the TRAMP mouse model (Figure 1C & 1D); initiated with hyperplasia (6-10 weeks), then progressing through low-grade prostate intraepithelial neoplasia (PIN) (10-14 weeks), high-grade PIN (14-18 weeks), to prostate adenocarcinoma (18-22 weeks). The most advanced proliferative lesion in each lobe signified the grade of disease. Representative images (H&E-stained sections) of the various stages of disease severity (grades) can be seen in Figure 1D.

Inductively coupled plasma mass spectrometry (ICP-MS) was used to establish whether the concentration of copper, and of other metals (e.g. zinc and iron), changed in the prostate lobes of TRAMP mice during disease progression (Figure 2). At no stage during prostate cancer development (6, 10, 14, 18 and 22-weeks of age) did copper levels fluctuate in comparison to wt mice (Figure 2A–2C). These results are analogous to our recent findings with human patient prostatectomy samples [26], where the vast majority of patients regardless of disease severity (Gleason Score 7 or 9) had intratumoral copper levels within the normal range. Additionally, serum copper level measured across disease progression in TRAMP mice did not change (Figure 2D), as likewise noted in human prostate cancer patients [26]. In contrast, zinc levels were significantly lower in the ventral prostates of 18 and 22-weeks old TRAMP mice (Figure 2G), which is the first prostate lobe where adenocarcinoma arises in this model (Figure 1C) [35]. The zinc level in the other prostate lobes (anterior and dorsolateral), and in serum, did not vary between TRAMP and wt mice at any stage (Figure 2E, F & 2H). These findings are consistent with those by Costello and colleagues (2004 & 2011), who established that as in human prostate cancer, zinc levels are markedly decreased in TRAMP adenocarcinomas [33, 36]. The level of iron in anterior and dorsolateral prostate lobes was also significantly reduced in TRAMP mice at 22 weeks of age (Figure 2I & 2J), consistent with lower iron accumulation observed in prostate cancer patients compared to healthy subjects [37]. Note that serum iron could not be reliably measured due to varying degrees of hemolysis between samples, as ruptured erythrocytes change iron concentrations (not shown). There were no changes to the concentrations of other biologically relevant metals, such as selenium and manganese, in prostate tissues or serum (not shown). Taken together, these results position TRAMP mice as being a clinically relevant model to investigate metal homeostasis during prostate transformation to malignancy. Moreover, the significant copper-ionophore [Cu^II^(gtsm)] utility previously observed in this model [8] could not be explained by predisposing high intratumoral copper levels.

### Copper-ionophores induce oxidative stress in TRAMP prostate cancer cells

**Figure 3:**
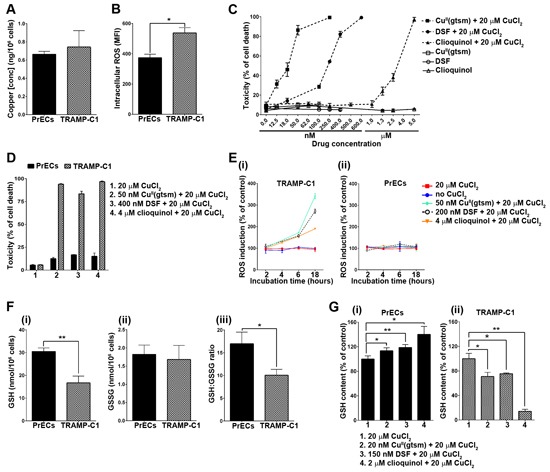
Copper-ionophores generate intracellular ROS and selectively target TRAMP adenocarcinoma cells through a disparity in their antioxidant capacity **A.** TRAMP adenocarcinoma cells (TRAMP-C1) have normal intracellular copper levels. Total intracellular copper was measured in both TRAMP-C1 and mouse primary prostate epithelial cells (PrECs) cultured under basal conditions. Results are shown as copper (ng) per 10^6^ cells. **B.** TRAMP adenocarcinoma cells (TRAMP-C1) have elevated intracellular ROS levels. Intracellular ROS was measured using the cell permeable fluorogenic probe H_2_DCF-DA and flow cytometry. Results represent mean fluorescence intensity (MFI) (geometric mean). **C.** Copper-ionophores potently kill TRAMP adenocarcinoma cells (TRAMP-C1). TRAMP-C1 cells were treated for 18 hours with Cu^II^(gtsm), disulfiram (DSF) or clioquinol alone or in combination with 20 μM CuCl_2_. Ionophore concentrations are shown and cell viability was determined by the propidium iodide exclusion assay and flow cytometry. **D.** Copper-ionophores selectively kill TRAMP adenocarcinoma cells while not affecting the viability of mouse primary prostate epithelial cells (PrECs). Both cell lines were treated for 18 hours with Cu^II^(gtsm), disulfiram or clioquinol in combination with 20 μM CuCl_2_. Ionophore concentrations are shown and cell viability was determined by the propidium iodide exclusion assay and flow cytometry. **E.** Copper-ionophores generate intracellular ROS in TRAMP adenocarcinoma cells (TRAMP-C1) **(i)**, but not in mouse primary prostate epithelial cells (PrECs) **(ii)**. Both cell lines were treated for 2, 4, 6 or 18 hours with Cu^II^(gtsm), disulfiram or clioquinol in combination with 20 μM CuCl_2_. Ionophore concentrations are shown and intracellular ROS was measured using the cell permeable fluorogenic probe H_2_DCF-DA and flow cytometry. Results represent mean fluorescence intensity (MFI) (geometric mean) **F.** TRAMP adenocarcinoma cells (TRAMP-C1) have markedly reduced antioxidant capacity. Reduced (GSH) **(i)** and oxidised (GSSG) **(ii)** glutathione were measured in TRAMP adenocarcinoma cells (TRAMP-C1) and mouse primary prostate epithelial cells (PrECs) by HPLC. **(iii)** The GSH:GSSG ratio is compared between both cell lines. **G.** Differential GSH expression in TRAMP adenocarcinoma cells (TRAMP-C1) treated with copper-ionophores. Reduced glutathione (GSH) was measured in mouse primary prostate epithelial cells (PrECs) **(i)** and TRAMP adenocarcinoma cells (TRAMP-C1) **(ii)** following treatment for 18 hours with sublethal concentrations of Cu^II^(gtsm) (20 nM), disulfiram (150 nM) or clioquinol (2 μM) (with 20 μM CuCl_2_). Glutathione (GSH & GSSG) was measured by HPLC. Results represent mean ± STDEV (bar) of triplicate determinations for each measurement. (*p < 0.05; **p < 0.01).

Three distinct copper-ionophores, Cu^II^(gtsm) [bis(thiosemicarbazone) analog], disulfiram (dithiocarbamate analog) and clioquinol (hydroxyquinoline analog), all release coordinated copper under the reductive intracellular environment [8] and display selective anticancer activity *in vitro* and in mouse models (reviewed in [9]). The differential response between healthy (normal) and cancerous cells to these copper-ionophores is the premise for their further development and conceivably, is due to a disparity in handling and detoxifying elevated prooxidant copper. To investigate this possibility, we compared prostate epithelial cells (PrECs) derived from wt mice to cancerous prostate epithelial cells (TRAMP-C1) derived from TRAMP mice (Figure 3). Prostate tissue used to establish the TRAMP-C1 cell line was obtained from a 32-week old TRAMP mouse bearing adenocarcinoma, as previously described [38]. The PrEC and TRAMP-C1 cell lines had comparable levels of intracellular copper (Figure 3A), consistent with our observations using *ex vivo* prostate tissues derived from wt and TRAMP mice (Figure 2A-2C). Nevertheless, TRAMP-C1 cells had markedly elevated levels of intracellular ROS (measured with H_2_DCF-DA probe) (Figure 3B), which is a well-defined feature in both human [22] and TRAMP prostate cancers [39]. Additionally, TRAMP-C1 cells were remarkably more sensitive in comparison to prostate epithelial cells (PrECs) to copper-ionophore treatments [Cu^II^(gtsm), disulfiram & clioquinol] (Figure 3C & 3D), indicating a large therapeutic window. Note that the proliferation of PrECs during the copper-ionophore treatments was unaffected (not shown). These results are consistent with our previous finding using human cells, where Cu^II^(gtsm) selectively killed prostate hyperplasia and carcinoma cell lines (BPH-1, PC3, DU145 & LNCaP), while not affecting the viability or proliferation of primary prostate epithelial cells [8]. To be consistent with our previous studies [7, 8], copper-ionophore treatments were in media supplemented with a physiological level in copper (20 μM CuCl_2_). Milieu copper is required for the anticancer activities of both disulfiram (DSF) and clioquinol ligands [7, 8]. Taken together, these results provide further evidence that intracellular copper status does not govern cellular sensitivity to copper-ionophores.

To establish if copper-ionophore toxicity correlated with oxidative stress, we compared both ROS production (Figure 3E) and antioxidant capacity (Figure 3F) between the PrEC and TRAMP-C1 cells. Strikingly, copper-ionophore treatments [Cu^II^(gtsm), disulfiram & clioquinol] caused ROS production only in TRAMP-C1 cells (Figure 3E[i] & 3[ii]). ROS was measurably elevated after 6 hours of treatment and was considerably augmented after 18 hours of treatment. The same copper-ionophore concentrations were associated with TRAMP-C1 cell death following 18 hours of treatment (Figure 3C & 3D). Glutathione (GSH) is the major cellular antioxidant and plays an essential role in protecting cells against ROS accumulation and toxicity [40, 41]. An imbalance in cellular GSH homeostasis caused by either GSH oxidation (to GSSG), or by GSH efflux, can diminish the antioxidant capacity of the cell and often contributes to cell death [40–42]. We determined the GSH:GSSG ratio in both PrEC and TRAMP-C1 cells, as a well-established marker for antioxidant capacity (Figure 3F[iii]) [40, 41]. The basal GSH:GSSG ratio was significantly lower in TRAMP-C1 cells than in PrEC cells (Figure 3F[iii]), meaning that there is less protective GSH (Figure 3F[i]) and more oxidized GSSG (Figure 3F[ii]) in the cancerous cells. Furthermore, sublethal copper-ionophore treatments [Cu^II^(gtsm), disulfiram & clioquinol] stimulated an increase in the level of protective GSH in PrEC cells (Figure 3G[i]), while in stark contrast, caused a reduction in GSH in TRAMP-C1 cells (Figure 3G[ii]). These results can account for there being ROS production observed only in TRAMP-C1 cells following copper-ionophore treatments (Figure 3E[i] & 3E[ii]). Taken together, these data demonstrate that adenocarcinoma TRAMP-C1 cells have diminished capacity to handle and detoxify auxiliary ROS compared to wt prostate epithelial cells (PrECs), providing a premise for how copper-ionophores are selective toward prostate cancer cells.

### Altered systemic copper distribution impedes prostate cancer growth

**Figure 4:**
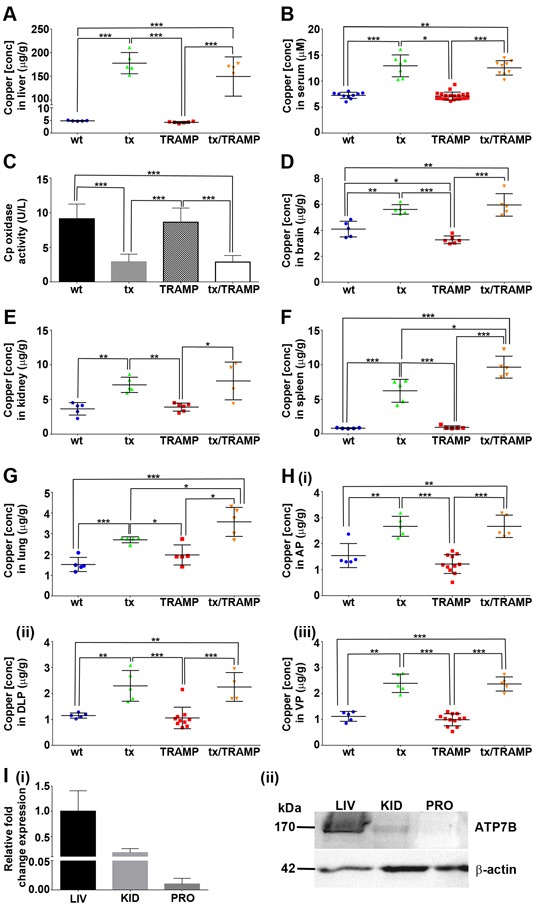
The tx mutation in Atp7b substantially alters systemic copper distribution in mice **A**&**B.** TRAMP mice harbouring the tx mutation (tx/TRAMP) display elevated copper in their liver and serum. Inductively coupled plasma mass spectrometry (ICP-MS) was used to determine copper concentrations in the liver and serum of 22-week old wild type, tx, TRAMP and tx/TRAMP mice (n=5-20 for each strain). Results represent mean ± STDEV (whisker plot) and are shown as either μg/g wet weight for tissue or μM for serum. **C.** Copper incorporation into serum ceruloplasmin is perturbed in TRAMP mice harbouring the tx mutation (tx/TRAMP). Serum ceruloplasmin oxidase activity (copper-dependent) was measured from 22-week old wild type (n=10), tx (n=11), TRAMP (n=17) and tx/TRAMP (n=10) mice using the *o*-dianisidine dihydrochloride based assay. Results are expressed as unit/litre (U/L) and presented as mean ± STDEV (bar). **D-H.** TRAMP mice harbouring the tx mutation (tx/TRAMP) display elevated copper in extrahepatic tissues. ICP-MS was used to determine copper concentrations in brain, kidney, spleen, lung and prostate lobes [anterior prostate (AP), dorsolateral prostate (DLP) and ventral prostate (VP)] of 22-week old wild type, tx, TRAMP and tx/TRAMP mice (n=4-20 for each strain). Results represent mean ± STDEV (whisker plot) and are shown as μg/g wet weight. **I.** Mouse prostate has no detectable level of Atp7b expression. (i) Real-time PCR quantification of *Atp7b* mRNA levels in liver (LIV), kidney (KID) and prostate (PRO) of 22-week old wild type mice (n=3). The level of *Atp7b* mRNA is compared against the liver. (ii) Western blot analysis of Atp7b expression in liver (LIV), kidney (KID) and prostate (PRO) of 22-week old wild type mice (50 μg protein). The WND4B antibody detected Atp7b in the liver and kidney at ~170kDa. β-actin was detected as a loading control. (*p < 0.05; **p < 0.01; ***p < 0.001).

**Figure 5:**
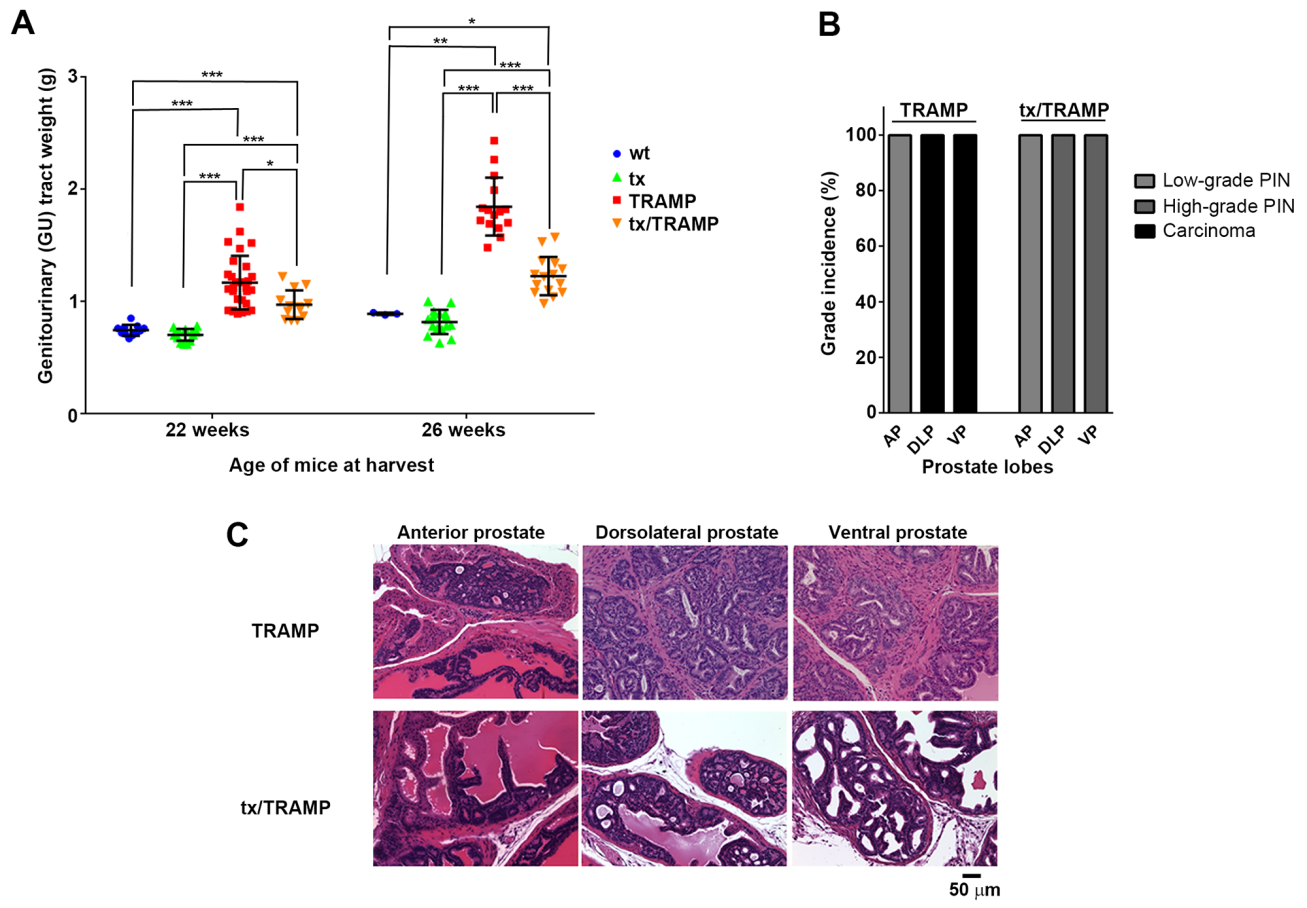
Altered systemic copper distribution impedes prostate cancer growth in TRAMP mice **A.** The tx mutation significantly reduces prostate cancer burden in TRAMP mice. Genitourinary (GU) tracts were weighed from 22-week old and 26-week old wild type, tx, TRAMP and tx/TRAMP mice and normalised to respective mouse body weights (n=5-15 for each strain). The mean ± STDEV (whisker plot) for each strain is shown. **B.** The tx mutation significantly reduces prostate cancer disease severity (grade) in TRAMP mice. Histological examination of prostate lobes [anterior prostate, dorsolateral prostate and ventral prostate] with hematoxylin and eosin (H&E) staining, establishing disease grade in 26-week old wild type, tx, TRAMP and tx/TRAMP mice (n=5 for each strain). The most advanced proliferative lesion in each lobe signified the grade of disease. **C.** Representative H&E-stained sections displaying prostate disease grade in the prostate lobes [anterior prostate (AP), dorsolateral prostate (DLP) and ventral prostate (VP)] of 26-week old TRAMP and tx/TRAMP mice. (*p < 0.05; **p < 0.01; ***p < 0.001)

Despite intratumoral copper levels being normal in the vast majority of prostate cancer patients [26], and in the TRAMP mouse model (Figure 2A, B & 2C), we wanted to establish whether copper is nevertheless important for prostate cancer growth. However, manipulating diet to regulate systemic copper levels in mice is extremely difficult, as mice are proficient at maintaining strict homeostatic copper levels [12, 43]. Therefore, we decided to instead alter copper supply to the prostate by genetically modifying copper release from the liver; the central organ controlling systemic copper distribution. The toxic milk mouse (tx), a well-characterised model for Wilson's disease, has an autosomal recessive mutation in the *Atp7b* gene (A4066G/Met1356Val) that causes substantial changes to systemic copper distribution [44, 45]. The gene encodes the copper-transporting P-type ATPase expressed primarily in the liver (Atp7b), which mediates both copper incorporation into serum components (systemic distribution) and the excretion of excess copper into bile [46]. The tx mutation in *Atp7b* hinders both of these functions [44, 45], therefore we assessed whether it could influence prostate cancer growth in the TRAMP mouse model (Figure 4 & 5).

The original tx mouse was on the inbred DL strain [44, 45, 47, 48], while the tx mice used in this study had been backcrossed to the C57BL/6 background for 10 generations. Therefore, we first verified that our tx C57BL/6 model retained the same characteristics (Figure 4). Consistently, tx C57BL/6 mice (22-weeks old) accumulated substantial levels of hepatic copper (~35-fold increase) (Figure 4A) and had elevated serum copper content (~1.8-fold increase) (Figure 4B) [44, 47, 48]. Likewise, TRAMP mice harbouring the tx mutation (22-weeks old) had equivalent copper manifestations (Figure 4A & 4B). To substantiate that copper integration into serum components was also perturbed due to the tx mutation, we measured ceruloplasmin (CP) oxidase activity (Figure 4C). Ceruloplasmin coordinates 6 atoms of allosteric copper that are supplied during its biosynthesis by Atp7b (in hepatocytes) and accounts for more than 70% of the copper found in blood (serum) [9]. Consistent with the lack of fully functional Atp7b, mice harbouring the tx mutation (tx and tx/TRAMP) had significantly reduced serum ceruloplasmin oxidase activity, as measured with the conventional *o*-dianisidine dihydrochloride substrate (Figure 4C). A further feature of tx mice is copper dyshomeostasis in extrahepatic tissues [47, 48], presumably due to the altered serum copper content. Elevated copper was found to occur in all extrahepatic tissues examined, even those that are not believed to express *Atp7b* [47, 48]. We confirmed that mice harbouring the tx mutation (tx and tx/TRAMP) have elevated copper in their brain (1.3 to 1.8-fold increase) (Figure 4D), kidneys (2-fold increase) (Figure 4E) and spleen (7.5 to 12-fold increase) (Figure 4F) and further established the same dyshomeostasis in lungs (1.4 to 2.2-fold increase) (Figure 4G) and the prostate lobes (1.8 to 2.4-fold increase) (Figure 4H[i-iii]). Importantly, we demonstrated that normal prostate (wtC57BL/6), analogous to most extrahepatic tissues, does not express *Atp7b* at the mRNA (Figure 4I[i]) or protein level (Figure 4I[ii]), reducing the possibility of a direct influence of the tx mutation on prostate cancer growth. Therefore, together these results demonstrate that the tx mutation significantly alters systemic copper distribution, providing a unique model to explore the importance of copper in prostate cancer development and progression.

The impact of the tx mutation and associated systemic copper misdistribution on prostate cancer growth was assessed in 22 and 26-week old male TRAMP mice (Figure 5). These ages represent when TRAMP mice first develop adenocarcinoma (Figure 1) and when considerable prostate cancer growth had occurred (Figure 5A), respectively. Notably, there was no difference in GU tract weight between tx and wt mice at both 22 and 26-weeks of age (Figure 5A), demonstrating that the tx mutation does not alter normal prostate size. TRAMP mice exhibited prostate cancer burden across both age groups, with GU tract weights increasing 1.6–fold at 22 weeks and 2–fold at 26-weeks, when compared to age-matched wt mice (Figure 5A). Remarkably, TRAMP mice harbouring the tx mutation had significantly less prostate cancer burden in comparison to the standard TRAMP mice. The difference was more pronounced at 26-weeks of age, where there was a staggering 64% reduction in prostate cancer weight (GU tract weight). Histological examination of prostate lobes was further used to establish the severity of the proliferative lesions in the 26-week old mice, signifying the grade of the disease (Figure 5B). In TRAMP mice harbouring the tx mutation there was a significant reduction in the severity of prostate lesions in both dorsolateral (DLP) and ventral (VP) lobes, with no distinguishable adenocarcinoma. Representative images (H&E-stained sections) of the mean histological lesion seen in each lobe, for both TRAMP and tx harbouring TRAMP mice, can be seen in Figure 5C. Together, these results demonstrate that altering systemic copper, and thus copper supply to the prostate, significantly impedes prostate cancer growth and reduces disease severity in TRAMP mice.

## DISCUSSION

Copper-ionophores are being appraised for a variety of therapeutic indications that require a wide range of copper-related pharmacological effects. Clioquinol analogues (e.g. PBT2) are being trialled in Alzheimer's disease patients, having been shown to dissolve senile plaques (pathological hallmark) in mice by redistributing copper away from amyloid and into neighbouring neurons [49–51]. Similarly, Cu^II^(atsm) [diacetylbis(*N*^4^-methylthiosemicarbazonato)Cu^II^] has proven to be an effective treatment for mice with motor neuron disease (ALS) [52]. Cu^II^(atsm) releases coordinated copper in elevated reductive states [e.g. hypoxic tissues, electron transport chain (ETC) compromised cells] [8, 53, 54] and corrected superoxide dismutase (SOD) (cuproenzyme) levels in copper deficient spinal cords of ALS mice [52, 54]. Remarkably, ALS mice treated with Cu^II^(atsm) had 18-months of extended survival [52]. These promising studies demonstrate the importance of understanding biological and pharmacological activities of copper-ionophores for their therapeutic purpose. Despite extensive study, there is inadequate understanding of how copper-ionophores function as anticancer agents. In this study, we delineated the role of elevated intratumoral copper and ROS and provided a premise for how copper-ionophores are selectively toxic toward prostate cancer cells.

Prostate tissue copper levels in TRAMP mice remained unchanged during prostate cancer development and progression (Figure 2A–2D) and thus could not explain the significant copper-ionophore [Cu^II^(gtsm)] utility previously observed in this model [8]. Cultured TRAMP mouse adenocarcinoma cells (TRAMP-C1) were likewise susceptible to copper-ionophore treatment [Cu^II^(gtsm), disulfiram & clioquinol] (Figure 3C & 3D), despite having normal intracellular copper levels when compared to treatment-refractory mouse prostate epithelial cells (PrECs) (Figure 3A). These findings are consistent with the vast majority of patients with prostate cancer (Gleason Score 7 & 9) having intratumoral copper levels well within the normal range [26]. Enhanced copper uptake by prostate cancer cell lines *in vitro* and in xenografts mouse models, mediated by copper transporter 1 (hCtr1) protein, elevates intracellular copper and has been suggested to underpin copper-ionophore anticancer activity [17, 18, 23]. Our results establish that surplus copper does not govern the pharmacological facility of copper-ionophores, but may heighten clinical activity in the small subset of patients found to have elevated intratumoral copper [26]. Additionally, surplus copper is not required for the altered metabolic requirement of prostate cancer. Consistent with malignant transformation causing a loss of cell specialization, zinc levels were found to be significantly lower following adenocarcinoma onset in TRAMP mice (ventral lobe) (Figure 2G) [33, 36]. Healthy prostate (includes BPH) contains an extremely high concentration of zinc, more than any other soft tissue in the body (Figure 2E–2H) [55] and requires zinc to change metabolism in order to produce citrate, an important component of semen. Prostate cancer malignancy necessitates the reduction of intracellular zinc to activate mitochondrial aconitase and in turn induce citrate oxidation and ATP production (Krebs cycle) [33, 36, 56]. Accordingly, zinc-ionophores have recently emerged as potential therapeutics for prostate cancer [57]. Together, copper and zinc levels in TRAMP mice mirror those seen in patients with prostate cancer (tissue and serum) (Figure 2A–2H) [33, 36, 57], positioning TRAMP mice as being clinically relevant to investigate ionophore pharmacology.

We demonstrated that TRAMP adenocarcinoma cells (TRAMP-C1) have markedly reduced capacity to detoxify elevated prooxidant (ionophoric) copper (Figure 3E & 3F), providing a basis for their selective death *in vitro* (Figure 3C & 3D) and in mice [8] following copper-ionophore treatment. A hallmark of the aggressive phenotype of prostate cancer is oxidative stress (increased ROS), which has been linked to disease development and progression [58–60]. Clinical trials utilizing antioxidants have been disappointing (reviewed in [60]), and conceivably prooxidant agents (e.g. ionophoric copper) used to instead overload the cells with oxidative stress may prove more effective. Copper-ionophores [Cu^II^(gtsm), disulfiram & clioquinol] that release coordinated copper within cells [8] catalysed the formation of intracellular ROS in TRAMP adenocarcinoma cells (TRAMP-C1), but not in normal prostate epithelial cells (PrECs) at the same concentrations (Figure 3E). The anticancer activities of Cu^II^(gtsm) and disulfiram have previously been attributed to ROS production, as antioxidants (e.g. *N*-acetyl-L-cysteine) attenuate their effectiveness [23, 61]. Ordinarily, cells have potent antioxidant defence mechanisms and one key player, glutathione [reduced (GSH) or oxidised (GSSG)], serves as an indicator of the cellular redox status [40]. Glutathione (GSH) can directly donate an electron (reducing equivalent; H^+^+ e^−^) to unstable molecules, such as ROS. In turn glutathione itself becomes reactive, but rapidly bridges (disulfide) with another reactive glutathione to form the corresponding disulfide (GSSG). Protein glutathionylation can also increase under oxidative stress and protects thiols from oxidative modifications that can irreversibly alter protein function or stability [62]. However, protein glutathionylation is dependent on cellular redox status, typically occurring only following substantial disruption to the GSH:GSSG ratio [63]. The capacity of cells to buffer oxidative stress depends on the GSH:GSSG ratio and a reduced intracellular GSH level, through oxidation or cellular efflux, has been associated with cell death induction [40, 41]. There have been several reports that glutathione activity is perturbed in prostate cancer [64, 65] and indeed treatment with L-buthionine-sulfoximine (BSO), an inhibitor of glutathione synthesis, potentiates prooxidant (As_2_O_3_) toxicity in prostate cancer cells [66]. We demonstrated that TRAMP adenocarcinoma cells (TRAMP-C1) have markedly elevated ROS (Figure 3B) coupled with a reduced basal GSH:GSSG ratio that corresponded to considerably less protective GSH (~50%) (Figure 3F[I]-[III]). Furthermore, copper-ionophore [Cu^II^(gtsm), disulfiram & clioquinol] treatments further decreased GSH levels in the prostate cancer cells (Figure 3G[ii]), indicative of exceeding their antioxidant capacity. Conversely, identical treatments of normal prostate epithelial cells (PrECs) increased their intracellular GSH level (Figure 3G[i]), thereby affording protection from oxidative stress-induced cell death. Taken together, our findings clarify that the anticancer activity of copper-ionophores is not reliant on intracellular copper level, but rather on a disparity in cellular ROS and antioxidant capacity.

Wild type mice are proficient at maintaining strict homeostatic copper levels and are resistant to copper loading through diet [12, 43]. The liver sequesters newly absorbed dietary copper and through Atp7b regulates both copper incorporation into serum components (systemic distribution) and biliary excretion of superfluous copper [46]. The tx mutation (Met1356Val) obstructs the copper-translocation activity of Atp7b [67], causing hepatic copper accumulation and altered systemic copper distribution [43–45, 47]. The tx mouse is a well-established model for the hepatic manifestations seen in Wilson disease and exhibits overt liver pathology at around 10-months of age [68]. Haplosufficiency necessitates that both *Atp7b* alleles are mutated [43]. An intriguing feature of tx mice is copper being systemically elevated in extrahepatic tissues (brain, kidney, spleen, lung & prostate) (Figure 4D–4H) [43], even in tissues believed not to express Atp7b (lung & spleen). We confirmed that mouse prostate does not express Atp7b at the mRNA or protein level (Figure 4I). Therefore, the copper dyshomeostasis in extrahepatic tissues is seemingly caused by altered copper supply to these tissues. Copper distribution to peripheral tissues is poorly understood, but circulating ceruloplasmin is known to be the major copper-carrying protein (holds >70% of serum copper) [9]. The oxidase activity of ceruloplasmin requires copper metallation during its biosynthesis, which in hepatocytes is mediated by Atp7b [9]. We verified that copper integration into serum ceruloplasmin is impeded due to the tx mutation (Figure 4C), but nevertheless, tx mice harboured elevated serum copper levels (Figure 4B). How copper is alternatively incorporated into serum in tx mice is part of future investigations. We can confirm however, that ceruloplasmin deficiency alone is not responsible for the elevated extrahepatic tissue copper [69], having shown that ceruloplasmin knockout mice have no such copper aberration (Supplementary Figure S1). Therefore, the tx mutation more drastically alters systemic copper distribution, providing a unique model to explore the importance of copper in prostate cancer development and progression.

Our study is the first to show that prostate cancer growth is reliant on a functional copper supply from the liver (Figure 5). TRAMP mice aged to 26-weeks harbouring the tx mutation have a remarkable reduction in both prostate cancer burden (64% reduction) (Figure 5A) and disease severity (grade), with no discernable carcinoma development (Figure 5B). Copper was evidently more important for prostate adenocarcinoma development as appose to PIN development (low & high-grade), as 22-weeks old TRAMP mice harbouring the tx mutation showed less significant disease reduction (Figure 5A & 5B). Prostate cancer develops uniformly in the TRAMP mouse model, progressing through high-grade PIN to prostate adenocarcinoma at 18-22 weeks of age (Figure 1C). Surplus intratumoral copper is not a feature of prostate adenocarcinoma, in both patients (e.g. Gleason Score 7 & 9) [26] and TRAMP mice (across all grades) (Figure 2A–2C) and is thus not a requirement for the altered metabolic needs of aggressive malignancy. Instead, collectively our results demonstrate that prostate adenocarcinoma development requires the appropriate delivery of copper to the prostate gland, through a conventional serum carrier(s). Defining the important serum copper carrier(s) may provide opportunity to pharmacologically exploit this requirement. Moreover, understanding the pharmacological effects of complexes that target copper (e.g. chelators & ionophores) may better tailor their design and *in vivo* activities, which may include altering serum copper distribution. The tx mutation by altering serum copper distribution actually caused an increase in intratumoral copper in TRAMP mice (Figure 4H). Copper is required for numerous cuproenzymes involved in cancer progression and metastasis [9], however, the surplus intratumoral copper caused by altered delivery from the serum did not enhance prostate cancer progression. We also cannot rule out the possibility that the increased intratumoral copper impeded adenocarcinoma development.

In conclusion, we provided evidence that copper-ionophores can selectively target prostate cancer cells through a disparity in their antioxidant capacity and therefore are amendable for the treatment of patients with prostate cancer. Additionally, elevated intratumoral copper was not requisite for their anticancer activity and furthermore, was not associated with malignant transformation of the prostate in TRAMP mice as previously observed in patients [26]. We also demonstrated that prostate adenocarcinoma development requires a functional supply of copper, being significantly impeded by altered systemic copper distribution in mice. Our studies warrant further biological and pharmacological studies on copper-ionophores, aimed at tailoring their activities specifically for prostate cancer therapy.

## MATERIALS AND METHODS

### Cell culture and reagents

TRAMP-C1 mouse prostate cancer cells were derived from the TRAMP (transgenic adenocarcinoma of mouse prostate) strain (C57BL/6 background) [38] and were generously provided by Assoc. Prof. Michael H. Kershaw (Peter MacCallum Cancer Centre, Melbourne, Australia). Mouse (C57BL/6) primary prostate epithelial cells (PrECs) were purchased from Cell Biologics (Chicago, USA; Cat#C57-6038). TRAMP-C1 cells were cultured in DMEM (ThermoFisher, Scoresby, Australia; Cat#11965-092) supplemented with 10% foetal calf serum (Bovogen biologicals, Keilor East, Australia; Cat#SFBS-F), 2 mM L-glutamine, 100 Units/mL penicillin and 100 μg/mL streptomycin. Primary prostate epithelial cells were cultured in Epithelial Cell Medium (Cell Biologics, Chicago, USA; Cat# M6621) as per the manufacturer's instructions. Cells were maintained at 37°C under humidified atmosphere containing 5% CO_2_.

Copper-ionophores (Disulfiram; Cat#86720 and clioquinol; Cat#24880) were purchased from Sigma-Aldrich (Castle Hill, Australia) or synthesized [Cu^II^(gtsm)] by Assoc. Prof. Paul S. Donnelly (University of Melbourne, Melbourne, Australia) following published procedures [70]. Each copper-ionophore was prepared in DMSO at 5 mM immediately before each experiment. All other reagents were supplied by Sigma-Aldrich (Castle Hill, Australia) unless specified otherwise.

### Mouse experiments

Experiments were conducted in accordance with national and international guidelines and were reviewed and approved by the Deakin University Animal Ethics Committee (AEC) (G21/2013 & G01/2014). Homozygous TRAMP mice on the C57BL/6 background were maintained as previously described [8], while wild type (wt) C57BL/6 mice were purchased from the Australian Animal Resources Centre (Canning Vale, Australia). Ceruloplasmin (CP) knockout C57BL/6 mice and wt littermates were kindly provided by Dr. Scott Ayton (University of Melbourne, Australia). Heterozygous TRAMP males (referred to as TRAMP mice) were generated by crossing founder homozygous TRAMP males with wtC57BL/6 females. Toxic milk mice on the C57BL/6 background (referred to as tx mice), containing the A4066G/Met1356Val mutation in the Atp7b copper-ATPase [53], were obtained from Prof. Julian Mercer (Deakin University, Burwood, Australia). Several breeding steps were required to generate heterozygous TRAMP/homozygous tx male mice (Tt/XX; where T = Tramp & X = tx alleles). Initially, both homozygous mouse strains were crossed to produce heterozygous TRAMP/heterozygous tx progeny (Tt/Xx). The progeny (Tt/Xx) were then backcrossed with homozygous tx mice (tt/XX) to obtain both heterozygous TRAMP/homozygous tx male mice (Tt/XX) and homozygous tx male mice (tt/XX).

Blood was collected by cardiac puncture and incubated on ice for 30 min before serum was isolated by two centrifugations at 16,000 g for 5 min and then snap frozen. Genitourinary (GU) tracts (includes prostate, seminal vesicles, testicles and emptied urinary bladder) were removed and their weights normalized against respective mouse weights.

### Genotyping

The genotypes of all mouse strains were confirmed by PCR-based screening using DNA isolated from ear tags. The DNA was extracted using the Viagen DirectPCR® DNA Extraction System (Viagen, Los Angeles, USA; Cat#402-E) with 0.2 mg/mL proteinase K. PCR was performed using 2xGo Taq® Hot Start Green Master Mix (Promega, Alexandria, Australia; Cat#M5122) with the primer sets shown below. Presence of the TRAMP *SV40 T antigen* transgene was established using the forward 5′-CCGGTCGACCGGAAGCTTCCACAAGTGCATTTA-3′ and reverse 5′-CTCCTTTCAAGACCTAGAAGGTCCA-3′ primers as previously described [32]. Primers against mouse IL-2 were used as an internal PCR control (forward: 5′-CTAGGCCACAGAATTGAAAGATCT-3′ and reverse: 5′-GTAGGTGGAAATTCTAGCATCATCC-3′). Reactions were run on a Biometra PCR machine (Biolabo Scientific Instruments, Chatel-St-Denis, Australia) using an amplification program of 1 cycle at 94°C for 5 min, 40 cycles at 94°C for 30 s, 54°C for 30 s, 72°C for 1 min and 1 cycle at 72°C for 5 min. Primers used to amplify the relevant region of the *Atp7b* gene, to confirm the tx A4066G/Met1356Val mutation, were forward 5′-GAGCAGGGCTCTCAGTATTCCCTAGC-3′ (complementary sequence before exon 19) and reverse 5′-GGATACTGAATTCCCATGGTTCAAG-3′ (complementary sequence before exon 21). The program for amplifying the *Atp7b* region was 1 cycle at 96°C for 3 min, 36 cycles at 96°C for 1 min, 55°C for 1 min, 72°C for 1 min and 1 cycle at 72°C for 5 min. The resultant 659-bp PCR product was then digested with *Nco*I restriction endonuclease for 2 hours at 37°C. The attainable *Nco*I profiles included 4 fragments for wt*Atp7b* (103, 345, 193 and 18-bp), 3 fragments for the tx mutation (448, 193 and 18-bp) and 5 fragments for heterozygous mice (103, 345,448, 193 and 18-bp).

### Tissue, serum and intracellular metal analysis

Metal concentrations were determined by inductively coupled plasma mass spectrometry (ICP-MS). The prostate lobe and other organs [kidney, spleen, lung, liver and brain (left hemisphere)] were freeze-dried before being digested in 65% nitric acid (50-500 μL) (Suprapur, Merck, Bayswater, Australia; Cat#100441) overnight at room temperature and then at 90°C for 20 min. An equivalent volume of 30% hydrogen peroxide (VWR, Tingalpa, Australia; Cat#87003-224) was then added to each sample. Samples were incubated 30 min at room temperature and 15 min at 70°C and were further diluted with 1% nitric acid (900-1000 μl). Serum samples (50 μL) were diluted in 1% nitric acid (450 μL). Intracellular metal analysis of PrEC and TRAMP-C1 cells was performed as follows. Cellular pellets were generated as previously described [7] and to each pellet 50 μL of 65% nitric acid were added and the samples were incubated for 6 hours at room temperature before being heated at 90°C for 20 min. After digestion, 455 μL of 1% nitric acid were added to reach a final volume of 500 μL. All metal measurements were made using an Agilent 7700 series ICPMS instrument under routine multi-element operating conditions using a Helium Reaction Gas Cell. The instrument was calibrated using 0, 5, 10, 50, 100 and 500 ppb of certified multi-element ICPMS standard calibration solutions (AccuStandard, New Haven, USA; Cat#ICP-MS-CAL2-1, ICP-MS-CAL-3 & ICP-MS-CAL-4) for a range of elements and a certified internal standard solution containing 200 ppb of Yttrium (Y89) was used as an internal control (AccuStandard; Cat#ICP-MS-IS-MIX1-1). The raw ppb values obtained were converted into either μg/g of wet weight for tissues (μg/g), to μmol/L for serum, or to ng/10^6^ cells for tissue culture as previously described [26].

### Histological examination

Dissected prostate lobes were fixed in 10% neutral buffered formalin overnight at 4°C, before being transferred into 70% ethanol and paraffin-embedded. Sections were cut and stained with hematoxylin and eosin (H&E). Prostate samples were step sectioned and histopathology was determined from at least 6 sections per mouse using the TRAMP model scoring system as previously described [8]. The most aggressive histological lesion observed in each lobe was used to grade each lobe and therefore the severity of disease.

### Serum ceruloplasmin oxidase activity assay

The oxidase activity of ceruloplasmin in serum was measured spectrophotometrically by using *o*-dianisidine dihydrochloride (Sigma-Aldrich, Castle Hill, Australia; Cat#D3252) as a substrate, as previously described [71]. Briefly, in duplicate 96-well plates 5 μL of sera (or water for blank) was added to wells containing 75 μL of 0.1 M sodium acetate. After a 5 min-incubation at 37°C, 20 μL of 7.88 mM *o*-dianisidine dihydrochloride was then added to each well. One plate was incubated for 5 min and the other plate for 60 min at 37°C. The reaction was then stopped by the addition of 200 μL of 9 M sulphuric acid. After 5 min of incubation at room temperature, absorbance was read at 540 nm using a multiplate reader (Multiskan, ThermoFisher Scientific). Sera from each mouse strain (wt, tx, TRAMP & tx/TRAMP) was taken for at least 10 individual mice (n=10 to 17) and analysed in duplicate. Ceruloplasmin oxidase activity was expressed in International Units per litre (U/L) using the following formula: (U/L) = (((A_60min_-A_5min_)/55))/ε × 1/b × 60 × 1000), where A_60min_ and A_5min_ are the absorbance of the 60 min and 5 min solutions, respectively; ε is the molar absorptivity of coloured solutions in terms of substrate consumed (9.6 mL.μmol^−1^.cm^−1^); b = optical length (1 cm); 60 = volume correction factor and 1000 = conversion to 1L.

### Cell viability and intracellular ROS analysis

Mouse primary prostate epithelial cells (PrECs) and TRAMP-C1 cells were seeded (120,000 cells/well) separately into 24-well plates and incubated overnight. Cells were then treated for 2, 4, 6 or 18 hours in triplicate with various concentrations of Cu^II^(gtsm), disulfiram (DSF), clioquinol or Cu^II^(atsm) in complete medium with or without 20 μM CuCl_2_. Following treatment, conditioned media were collected in 5 mL FACS tubes and adhered cells were harvested using 1 mL of trypsin solution (0.025% trypsin and 0.02% EDTA) and then combined with their corresponding conditioned medium. Cells were pelleted by centrifugation at 1,500 rpm for 5 min at 4°C. To determine cell viability, cell pellets were resuspended in 300 μL of PBS containing 5 μg/mL propidium iodide (PI, excitation 538 nm/emission 617 nm) and immediately analysed with a FACS Canto II flow cytometer (BD Biosciences). 10,000 events were measured and the percentage of dead cells (cells positive for PI) was determined. To determine intracellular ROS levels following treatment, the cells were instead incubated in 500 μL of PBS containing 20 μM 2′,7′-dichlorodihydrofluorescein diacetate (H_2_DCF-DA) (ThermoFisher Scientific, Scoresby, Australia; Cat#D399) for 30 min at 37°C in the dark. Substrate reaction with ROS results in the production of fluorescent dichlorofluorescein (DCF) (excitation 495 nm; emission 527 nm). To concurrently determine cell viability, PI (2 μg/mL) was added to each tube immediately prior to measuring ROS. ROS levels were determined by comparing the mean fluorescence of treated versus control cells and were expressed as percentage of control. 10,000 events were measured and only viable cells (negative for PI) were used for analysis. Data analysis was performed with BD FACSDiva software version 8.0 (BD Biosciences).

### GSH and GSSG measurement

GSH and GSSG were measured by HPLC with permanganate chemiluminescence detection [72, 73] using a GloCel detector with serpentine flow-cell [74]. Cell pellets (1 × 10^6^ cells) were homogenised (vortexed) in 300 μL of 0.1% formic acid and then centrifuged at 13000 rpm for 15 min at 4°C. For GSH determination, the supernatant was diluted 10-fold in aqueous formic acid (5%) immediately prior to analysis. For GSSG determination, a second aliquot of supernatant (100 μL) was combined with 20 μL of 675 mM Tris-HCl buffer (pH 8.0) and 20 μL of 6.3 mM *N*-ethylmaleimide (NEM) to block endogenous GSH, and mixed for 30 s. Then 20 μL of 8 mM 2-mercaptoethanol was added to react excess NEM, and mixed for a further 30 s. To allow for complete disulfide bond reduction, 20 μL of 0.78 mM tris(2-carboxyethyl)phosphine hydrochloride (TCEP) was added and the solution gently heated at 50°C for 60 min. Finally, 20 μL of aqueous formic acid (5%) was introduced to re-acidify the sample prior to analysis.

### Western blot analysis

To detect Atp7b expression in mouse prostate, dissected lobes (AP, DLP and VP) were pooled together (per mouse) and homogenized as described previously [75]. Control organs, including the liver and kidney, were similarly prepared. Protein lysates were prepared by homogenizing approximately 20 mg of ground liver, kidney or prostate tissue, in buffer containing 50 mM Tris, 150 mM NaCl, 0.1% SDS, 0.5% sodium deoxycholate, 1% Triton-X 100, and complete mini-EDTA free protease inhibitor cocktail tablet (Roche Diagnostics GmbH, Castle Hill, Australia; Cat#04693124001). Protein samples (50 μg) were fractionated on a 7.5% SDS-PAGE and transferred to a 0.45 μm nitrocellulose membrane (GE Health Care Life Sciences, Australia; Cat#10600002) using the Mini Protean Tetra system (BioRad), before being blocked with 5% non-fat dry milk in TBS-T for 1 hour at room temperature. Mouse Atp7b was detected using mWND4B [44] (diluted 1:5,000), followed by HRP-conjugated donkey anti-goat secondary antibody (Sigma-Aldrich, Castle Hill, Australia; Cat#A5420) (diluted 1:5,000). Mouse ceruloplasmin was detected with rabbit anti-human ceruloplasmin (Dako, North Sydney, Australia; Cat#Q0121)) (diluted 1:1,000), followed by horseradish peroxidase-conjugated goat anti-rabbit secondary antibody (Dako Cat#P0448) (dilution 1:5,000). Anti-β-actin antibody (Sigma-Aldrich, Castle Hill, Australia; Cat#A5441) (diluted 1:10,000) was used as a loading control, with goat anti-mouse HRP secondary antibody (Dako; Cat#A5420) (diluted 1:5,000). Bands were visualized using Immobilon Western chemiluminescent detection kit (Merck Millipore, Bayswater, Australia; Cat#WBKLS0500) with the ChemiDoc MP Imaging system controlled by ImageLab software version 5.1 (Bio-Rad).

### Real-time quantitative PCR

Tissues were homogenized as previously described [75] and total RNA isolated using the Bioline Isolate II RNA Mini Kit according to manufacturer's instructions (Bioline, Alexandria, Australia; Cat#BIO-52072). cDNA was then synthesized from 1 ug of RNA using the SensiFAST cDNA Synthesis Kit (Bioline, Alexandria, Australia; Cat#BIO-65053) according to manufacturer's instructions. The specific primers were: mouse *Atp7b* 5′-GAGGGTCCACAGCCCTACAG and 5′-GCGGGTCCTATTGTCTGAAGTT; and mouse *GAPDH* 5′- TCACCACCATGGAGAAGGC and 5′- GCTAAGCAGTTGGTGGTGCA. Real time PCR was performed in triplicate on 20 ng of cDNA with the Power SYBR-Green PCR Master Mix (ThermoFisher Scientific, Scoresby, Australia; Cat#4367659) using the AB 7500 Real Time PCR System (Applied Biosystems). Primer efficiencies were taken into consideration when comparing relative amounts of mRNA to the liver using pyQPCR Software v0.9.

### Statistical analysis

Statistical significance was determined using Student *t* test or multiple *t* tests (Holm-Sidak method) with the GraphPad Prism software (Version 6.05). Unless otherwise stated, the data shown are means of at least triplicate determinations for each test condition with standard deviation (± STDEV).

## SUPPLEMENTARY FIGURE


