# Supplementary material for: Copper as a target for prostate cancer therapeutics: copper-ionophore pharmacology and altering systemic copper distribution
Source: Oncotarget. 2016 May 9;7(24):37064–80. doi: 10.18632/oncotarget.9245 (PMC5095059; doi:10.18632/oncotarget.9245)
Supplement: Supplementary file 1 [file oncotarget-07-37064-s001.pdf]

Copper as a target for prostate cancer therapeutics: copper-ionophore pharmacology and altering systemic copper distribution

SUPPLEMENTARY FIGURE

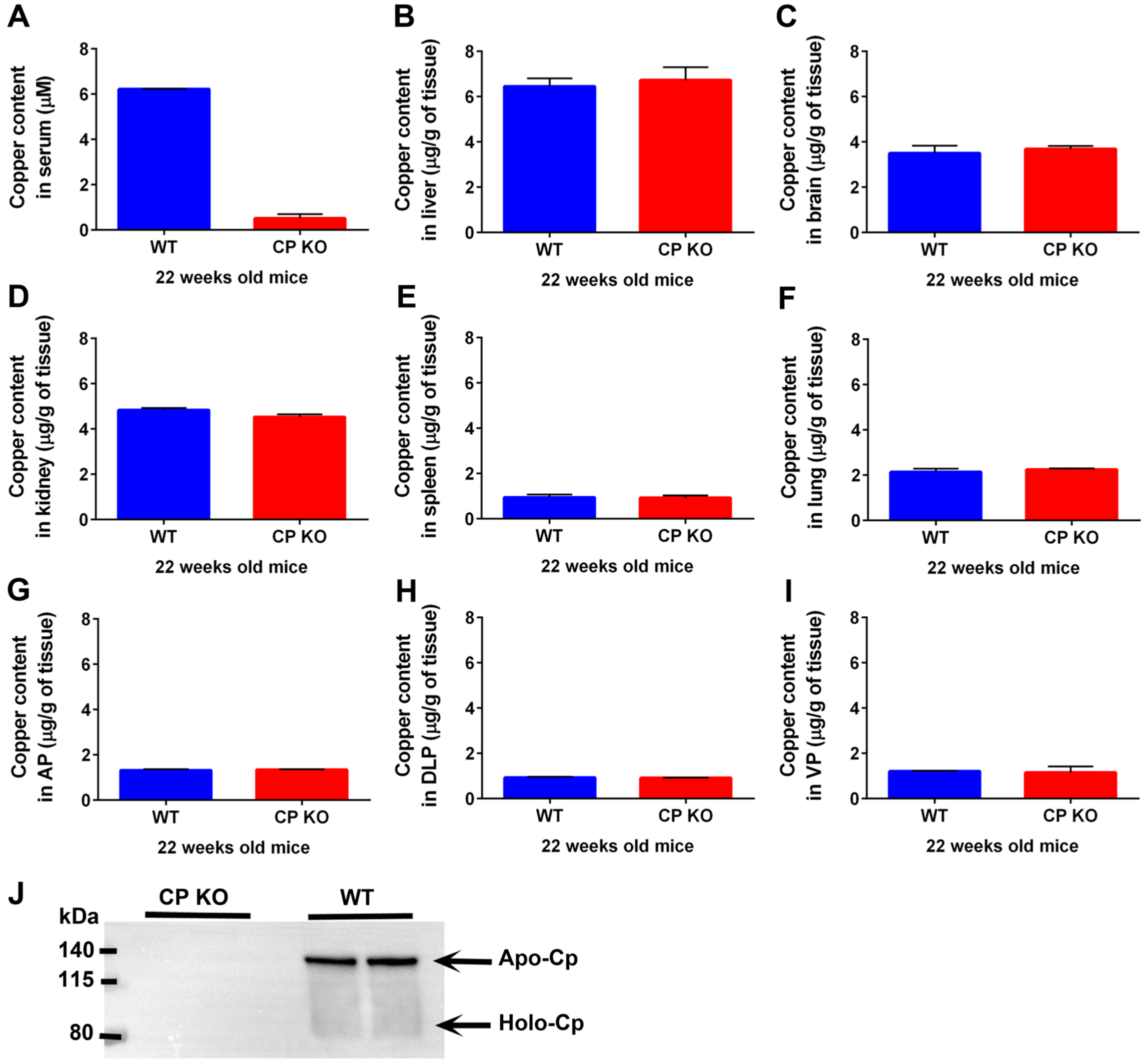

Supplementary Figure S1: Serum ceruloplasmin deficiency is not associated with copper dyshomeostasis in mice. Ceruloplasmin (CP) knockout mice have normal tissue copper levels. **A-I.** Inductively coupled plasma mass spectrometry (ICP-MS) was used to determine copper concentrations in serum, liver, brain, kidney, spleen, lung and prostate lobes [anterior prostate (AP), dorsolateral prostate (DLP) and ventral prostate (VP)] of 22-week old wild type (n=2) and ceruloplasmin knockout mice (n=2). Results are shown as either µg/g wet weight for tissues or µM for serum. **J.** Western blot analysis confirming Ceruloplasmin knockout. Western blot analysis of ceruloplasmin (CP) expression in serum of 22-week old wild type (n=2) and ceruloplasmin knockout mice (n=2) (50 µg protein).
